# Clinical Trial: Effect of Autologous Dendritic Cell Administration on Improving Neuropathy Symptoms and Inflammatory Biomarkers in Diabetic Neuropathy

**DOI:** 10.3390/cimb46120861

**Published:** 2024-12-20

**Authors:** Erwin Setiawan, Chrismis Novalinda Ginting, Jonny Jonny, Bhimo Aji Hernowo, Terawan Agus Putranto

**Affiliations:** 1 Faculty of Medicine, Dentistry, and Health Science, Universitas Prima Indonesia, Medan 20118, Indonesia; erwinsps2003@gmail.com (E.S.); chrismis@unprimdn.ac.id (C.N.G.); jonny@unprimdn.ac.id (J.J.); hernowobhimo@gmail.com (B.A.H.); 2 Department of Neurology, Gatot Soebroto Central Army Hospital, Jakarta 10410, Indonesia; 3 Faculty of Military Medicine, Indonesia Defence University, Bogor 16810, Indonesia; 4 Faculty of Medicine, Universitas Pembangunan Nasional “Veteran” Jakarta, Jakarta 12450, Indonesia; 5 Nephrology Division, Department of Internal Medicine, Gatot Soebroto Central Army Hospital, Jakarta 10410, Indonesia; 6 Indonesia Army Cellcure Center, Gatot Soebroto Central Army Hospital, Jakarta 10410, Indonesia; 7 Department of Radiology, Gatot Soebroto Army Central Hospital, Jakarta 10410, Indonesia

**Keywords:** diabetic neuropathy, autologous dendritic cells, Toronto Clinical Neuropathy Score, Transforming Growth Factor-β, Vascular Cell Adhesion Molecule-1

## Abstract

Type 2 diabetes mellitus (T2DM) is a global health concern, with diabetic neuropathy (DN) being a prevalent complication. Current DN treatments focus on blood glucose control and pain management, which show limited efficacy. This study explored the effects of autologous dendritic cell (DC) administration on improving DN symptoms. A quasi-experimental clinical trial was conducted on 28 DN patients at Gatot Soebroto Army Hospital. Patients received autologous DC administration, with their Toronto Clinical Neuropathy Score (TCNS), Transforming Growth Factor-β (TGF-β), and Vascular Cell Adhesion Molecule-1 (VCAM-1) levels measured before and at four weeks after treatment. The results show an average TCNS reduction from 8.93 to 7.5 (*p* < 0.001). TGF-β levels increased slightly from 41.16 ng/mL to 44.18 ng/mL (*p* > 0.05). VCAM-1 levels increased from 1389.75 ng/mL to 1403.85 ng/mL. Correlation analysis showed that TGF-β levels had a significant negative correlation with the TCNS (r = −0.353; *p* = 0.033) and VCAM-1 levels (r = −0.521; *p* = 0.002). Autologous DC administration significantly improves DN. While the changes in TGF-β and VCAM-1 levels were not statistically significant, their trends suggest that there was an anti-inflammatory effect. These findings highlight the potential of autologous DC therapy as a complementary approach to manage DN through inflammation reduction and nerve repair.

## 1. Introduction

Diabetic neuropathy (DN) is one of the common complications of Type 2 diabetes mellitus (T2DM) that has a high prevalence. In China, the prevalence of DN in T2DM patients has reached 67.6%, and is especially found in the elderly and in low-income and low-education groups [1]. In Indonesia, about 28% of T2DM patients have DN, with studies showing no significant association between duration of the diabetes and the incidence of neuropathy [2]. DN also accounts for 35% of microvascular complications, where risk factors such as advanced age play an essential role in their development [3,4]. This high prevalence of DN demonstrates the importance of early screening and managing risk factors to prevent further complications.

The Toronto Clinical Neuropathy Score (TCNS) is used as a clinical assessment instrument that assesses the severity of neuropathy, especially the symptoms and sensory deficits that are often the initial manifestations of DN [5]. The TCNS has been validated by various measures of neuropathy, such as sural nerve fiber density and electrophysiological parameters, showing significant correlations, which make it a reliable tool for diagnosing and staging diabetic neuropathy [6]. Electromyography (EMG) complements TCNS by providing objective data on nerve function and muscle activity, and it can detect nerve damage that is not clinically apparent [7]. The combination of TCNS and EMG improves the accuracy of diagnosis and is highly beneficial in managing diabetic neuropathy.

Current management for DN focuses on prevention via blood sugar control, lifestyle changes, and pain management. Based on the evidence, blood sugar control only reduces the relative risk of DN by 5–9% and must be accompanied by lifestyle changes for more effective prevention. Also, the few existing guidelines on DN pain management include it together with other neuropathic pain, leading to inconsistencies [8]. Therefore, there is a need to develop DN therapies that target its pathogenesis.

Diabetes causes nitric oxide (NO) deficiency; activates alternative metabolic pathways; results in the accumulation of glycation end products (AGEs), oxidative stress, and inflammation through inflammatory molecules; and increases the expression of pro-inflammatory cytokines. Chronic hyperglycemia exacerbates cytokine infiltration in vascular tissue, inhibiting neuronal repair [9]. Autologous dendritic cells (DCs) from the immune system serve as antigen-presenting and potential anti-inflammatory agents, with studies showing the benefits of autologous DCs in diseases such as arthritis and autoimmune disorders as well as their potential to relieve peripheral nerve inflammation [10,11,12,13]. This study aimed to determine the changes in TCNSs and the anti-inflammatory effects following autologous DC administration in DN patients.

## 2. Materials and Methods

### 2.1. Study Design

This study is a quasi-experimental clinical trial with a pre-test and post-test design that aims to evaluate changes in the TCNS in DN patients after autologous dendritic cell administration. The study was conducted at Gatot Soebroto Army Central Hospital (RSPAD GS) on a population of DN patients undergoing outpatient care at the Internal Medicine Polyclinic. The Health Research Ethics Committee of RSPAD GS approved the study under Ethical Approval No:102/VIII/KEPK/2024. Before the start of the study, all the subjects were given clear information about the purpose, procedures, and risks and benefits of this study. The subjects were allowed to ask questions, and after fully understanding, they signed a written informed consent form as a sign of their agreement to participate in this study. Research ethics were applied to protect the subjects’ rights during the clinical trial process.

### 2.2. Study Subjects

The study subjects consisted of DN patients who met the inclusion criteria and who were outpatients at the polyclinic of RSPAD GS during April 2024. The sampling technique used was consecutive sampling, in which subjects who met the criteria were included in the study sequentially until the minimum quota of 28 subjects was met. With this number of subjects, significant changes in TCNSs, which are used as the main indicator to measure the level of neuropathy in subjects, are expected to be detected.

Eligible participants had to (1) be adults over 18, (2) be willing to comply with all study procedures and to provide written informed consent, (3) be capable of adhering to the study’s protocols, (4) be in good physical and mental health, (5) meet the diagnostic criteria for Type 2 diabetes mellitus according to the PERKENI 2021 guidelines, and (6) their electromyography (EMG) results should be consistent with diabetic neuropathy.

Exclusion criteria included (1) recent immunosuppressive treatment, (1) non-diabetic neuropathy, (3) other types of diabetes, (4) pregnancy, (5) immunodeficiency (HIV, HCV, HBV), (6) ongoing invasive cancer treatment, (7) thromboembolism history, or (8) physical/mental disabilities hindering participation. Those unwilling to sign consent were also excluded.

### 2.3. Participant Characteristics

The recruitment process began with 3103 patient visits to the RSPAD Internal Medicine Polyclinic in April 2024. Among these, 276 patients were from the Endocrine Metabolic Diabetes Clinic, and 390 were from the Nephrology Clinic. Following a screening based on the inclusion criteria, 156 patients qualified, while 510 did not meet the inclusion criteria. Of the 156 eligible patients, 30 agreed to participate in the study, while 126 declined. Out of the 30 consenting participants, 29 proceeded to participate in the study; however, 1 was excluded due to meeting the exclusion criteria. During the study, 1 participant was lost to follow-up, resulting in a total of 28 subjects who completed the study and who were analyzed.

In total, 28 subjects satisfied both the inclusion and exclusion criteria and completed the entire study protocol. The average age of the participants was 61 years, with a gender distribution of 9 men (32%) and 19 women (68%). Hypertension was the most common comorbidity, affecting 26 participants (93%). Most participants fell into the overweight category, with a body mass index indicative of this in 35 subjects (50.7%). Additionally, a majority of participants, 20 individuals (71%), were using insulin (Table 1).

**Table 1:** Subjects’ baseline characteristics.

Baseline Characteristics		
**Number of subjects**		28
**Gender,** **n (%)**	Men	9 (32)
	Women	19 (68)
**Age,** **mean**		61 ± 9.5
**Comorbidities,** **n (%)**	Hypertension	26 (93)
	Heart disease	11 (39)
	Stroke	1 (4)
	Osteoarthritis	8 (8)
**BMI,** **n (%)**	Underweight	2 (7)
	Normal weight	8 (29)
	Overweight	13 (46)
	Obese	5 (18)
**Types of Anti-diabetics,** **n (%)**	Sulfonylurea	9 (32)
	Biguanide	7 (25)
	α-Glucosidase inhibitor	5 (18)
	DPP4 inhibitors	3 (11)
	SGLT2 inhibitors	4 (14)
	Insulin	20 (71)

### 2.4. Study Procedure

Each recruited subject participated in a 5-week clinical trial. The study began with a screening phase to confirm eligibility for participation. Once deemed eligible, subjects underwent a baseline examination, which included blood collection for DC preparation, serum collection for Transforming Growth Factor-β (TGF-β) and Vascular Cell Adhesion Molecule-1 (VCAM-1) measurement, EMG examination to confirm the diagnosis of DN, and TCNS assessment. Additional initial laboratory tests were also conducted at this stage. Seven days after the blood collection, each subject received a subcutaneous DC injection in the deltoid region. Finally, four weeks following DC administration, a follow-up assessment was conducted, including serum collection, TCNS measurement, and repeat laboratory tests.

### 2.5. Clinical and Laboratory Assessments

The TCNS is a widely recognized tool for assessing the severity of DN. The TCNS consists of 13 assessment points organized into three subsections: (1) Symptom Score—a subjective, patient-reported subsection that evaluates symptoms such as numbness, tingling, and pain, with each scored on a scale from 0 to 1, reflecting the presence or absence of specific symptoms; (2) Reflex Score—an objective, examiner-assessed score focusing on ankle reflexes that is scored from 0 to 2, where 0 represents normal reflexes, 1 indicates decreased reflexes, and 2 signifies absent reflexes; and (3) Sensory Score—also examiner-assessed, this subsection evaluates the patient’s sensory function, including responses to vibration, pinprick, and temperature sensations, with each scored from 0 to 1 based on the presence or absence of sensation in specific areas of the feet. The total TCNS ranges from 0 to 19, with higher scores indicating a greater severity of neuropathy, categorized into mild (1–8), moderate (9–12), and severe neuropathy (13–19).

In this study, a single trained physician conducted all the TCNS assessments to ensure consistency and to minimize inter-rater variability. To confirm the DN diagnosis and to objectively assess nerve function, EMG, a reliable diagnostic tool for detecting nerve damage and measuring nerve conduction, was also performed. Two senior neurologists conducted the EMG examinations, focusing on the sural nerve (sensory) and tibial nerve (motor). Both nerves were assessed through measurements of the motor nerve conduction velocity and amplitude.

For laboratory biomarker testing, blood samples were collected to measure TGF-β and VCAM-1 levels. Both biomarkers were quantified using sandwich enzyme-linked immunosorbent assay (ELISA) kits, which offer high sensitivity and specificity for detecting these proteins in serum. Additionally, fasting blood glucose levels for each participant were measured. Blood samples were collected after an overnight fast (at least 8 h) to ensure accurate fasting glucose levels.

### 2.6. Autologous DC Preparation

A sample of 40 milliliters of peripheral blood was drawn from each participant. From this sample, peripheral blood mononuclear cells (PBMCs) were isolated and then cultured with Granulocyte-Macrophage Colony-Stimulating Factor (GM-CSF) and Interleukin-4 (IL-4) over five days to generate immature DCs. These were further incubated with the antigen for two additional days to induce maturation of these cells. The number of dendritic cells administered varied for each participant depending on the yield of DCs obtained from their blood sample. No adjustments were made to standardize the cell count across participants. Instead, the entire DC preparation derived from each 40 mL sample was administered, resulting in a dose range of approximately 0.5 to 8 million DCs, depending on the individual yield.

### 2.7. Clinical Evaluation and Laboratory Testing

After the injection, an evaluation of the TCNS was performed in week 4. In addition to the laboratory evaluation, the detection of inflammatory biomarkers, TGF-β, and VCAM-1 was performed at baseline and at 4 weeks after injection to observe changes in the inflammatory response. This study aims to evaluate the effect of autologous dendritic cell intervention on the diabetic neuropathy condition through various clinical and laboratory parameters.

### 2.8. Statistical Analysis

The statistical analysis for this study employed a range of tests to thoroughly assess the data from multiple perspectives. First, the Kolmogorov–Smirnov test was applied to determine the normality of the data distribution. For data that followed a normal distribution, a paired *t*-test was conducted to examine differences before and after the intervention. For non-normally distributed data, the Wilcoxon signed-rank test was utilized to compare values before and after the intervention. Additionally, Pearson correlation analysis was used to explore relationships between key variables, specifically focusing on changes in the TCNS and inflammatory biomarkers. All statistical analyses were performed using IBM SPSS Statistics (version 22), with Microsoft Excel employed for data visualization.

## 3. Results

### 3.1. Changes in the TCNS

This study measured the TCNS before and after the intervention. The mean TCNS before the intervention was 8.93, with a standard deviation of 2.73. After the intervention, there was a decrease in the mean TCNS to 7.5, with a standard deviation of 3.03. A normality test was conducted using the Shapiro–Wilk test before analyzing the difference between the before- and after-intervention scores. The *p*-value for the TCNS data before intervention in the normality test was 0.753, and the *p*-value for the after-intervention data was 0.775, indicating that both data sets were normally distributed.

**Figure 1:**
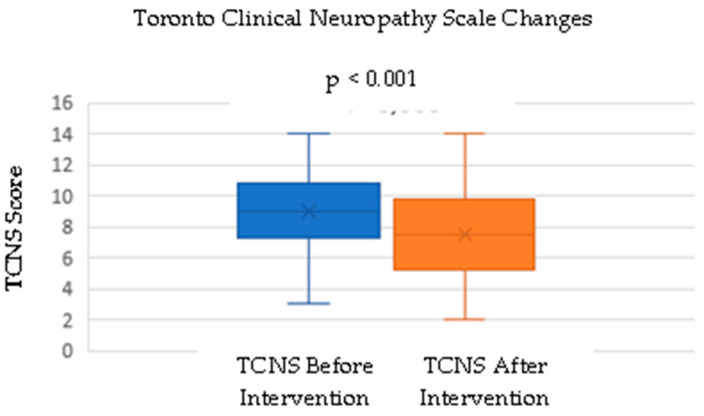
Change in the TCNS before and after DC administration.

**Figure 2:**
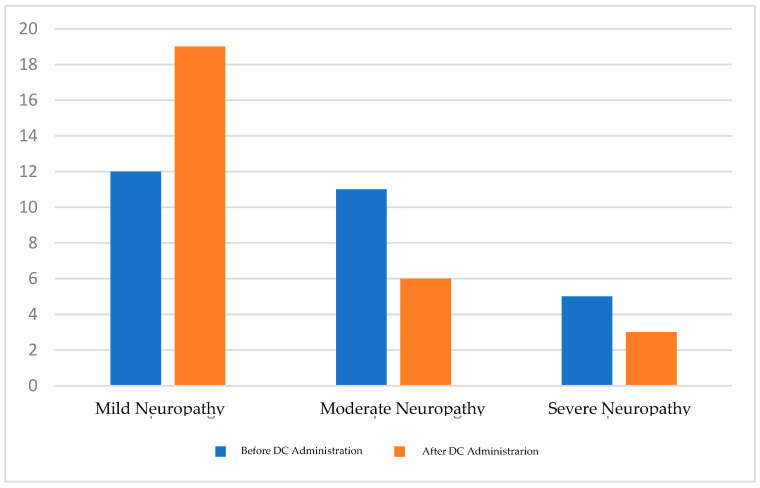
Diabetic neuropathy criteria shift.

A paired *t*-test was performed to test the hypothesis of a significant difference between the TCNSs before and after the intervention (Figure 1). The hypothesis test results showed a *p*-value of <0.001, indicating a statistically significant difference between the TCNSs before and after the intervention (Table 2). Additionally, the number of patients with mild neuropathy increased from 12 to 19, while the number of patients with severe neuropathy increased from 5 to 3 (Table 3; Figure 2).

**Table 2:** Changes in the TCNS, VCAM-1, and TGF-β.

Variables	Mean (Std. Deviation)	*p* Value Hypothesis Test
TCNS Before	8.93 (2.73)	<0.001
TCNS After	7.5 (3.03)	
VCAM-1 Before	1389.75 ng/mL (368.12)	0.101
VCAM-1 After	1403.85 ng/mL (410)	
TGF-β Before	41.16 ng/mL (11.83)	0.835
TGF-β After	44,18 ng/mL (15.25)	

Variables were measured before and at 4 weeks after DC administration. The normality test was performed using the Shapiro–Wilk test, while the hypothesis test was performed using the paired *t*-test. Abbreviations: Toronto Clinical Neuropathy Score (TCNS); Transforming Growth Factor-β (TGF-β); Vascular Cell Adhesion Molecule-1 (VCAM-1).

**Table 3:** Diabetic neuropathy criteria shift.

Diabetic Neuropathy Criteria (by the TCNS)	Before DC Administration	After DC Administration
Mild Neuropathy	12	19
Moderate Neuropathy	11	6
Severe Neuropathy	5	3

### 3.2. Effect on Fasting Blood Glucose

The results of hypothesis testing using a paired *t*-test showed no significant difference between fasting blood glucose levels before and after the intervention. The average fasting blood glucose level before the intervention was 143.07 ± 54.2 mg/dL, while 4 weeks after intervention, it was 143.46 ± 47.1 mg/dL, with a *p*-value of 0.970 (Table 4). This indicates that the intervention did not significantly affect fasting blood glucose levels.

**Table 4:** Change in fasting blood glucose.

Variables	Mean ± SD	*p*-Value Hypothesis Test
Fasting Blood Glucose Before	143.07 ± 54.2	0.970
Fasting Blood Glucose After	143.46 ± 47.1	

Variables were measured before and at 4 weeks after DC administration. The normality test was performed using the Shapiro–Wilk test; the hypothesis test was performed using the paired *t*-test.

### 3.3. Effect on VCAM-1 and TGF-β Levels

**Figure 3:**
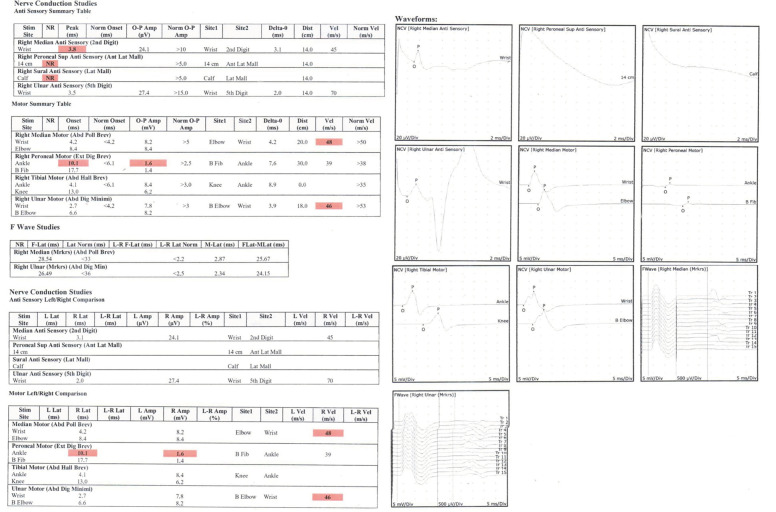
Example of an EMG study result. The red highlighted numbers means that the specific examination resulted in lower than normal value.

VCAM-1 levels increased slightly from a mean of 1389.75 ng/mL (SD = 368.12) before the intervention to 1403.85 ng/mL (SD = 410) after the intervention. A normality test using the Shapiro–Wilk method confirmed that both the before- and after-intervention data were normally distributed, with *p*-values of 0.419 and 0.169, respectively. A paired *t*-test was conducted to assess the significance of the change in VCAM-1 levels. The test yielded a *p*-value of 0.101 (Table 2; Figure 3), indicating that the increase in VCAM-1 levels was not statistically significant (*p* > 0.05). This suggests that the intervention did not have a significant impact on VCAM-1 levels, despite a small observed increase.

**Figure 4:**
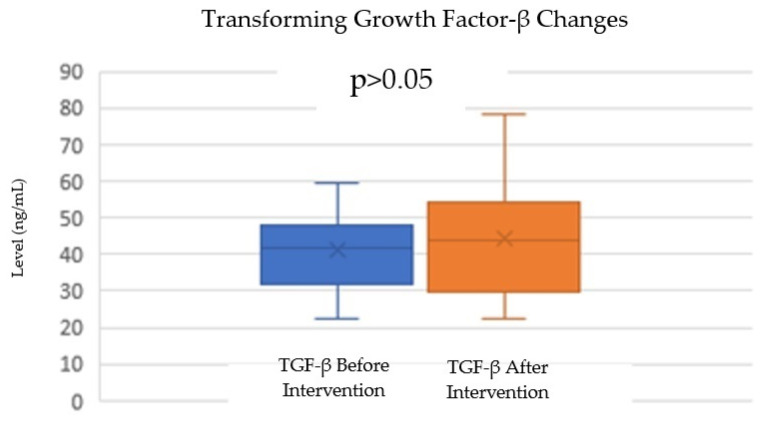
Change in TGF-β levels after autologous DC administration.

For TGF-β levels, there was a slight increase from a mean of 41.16 ng/mL (SD = 11.83) before the intervention to 44.18 ng/mL (SD = 15.25) after the intervention. The normality test for TGF-β levels indicated that both the before- and after-intervention data were normally distributed, with *p*-values of 0.524 and 0.835, respectively. A paired *t*-test was also conducted to evaluate the difference in TGF-β levels before and after the intervention. The test resulted in a *p*-value of 0.835 (Table 2; Figure 4), showing that the increase in TGF-β levels was not statistically significant (*p* > 0.05). While there was a trend toward increased TGF-β levels after the intervention, this change was not statistically significant.

### 3.4. Correlation of the TCNS and TGF-β and VCAM-1 Levels

**Figure 5:**
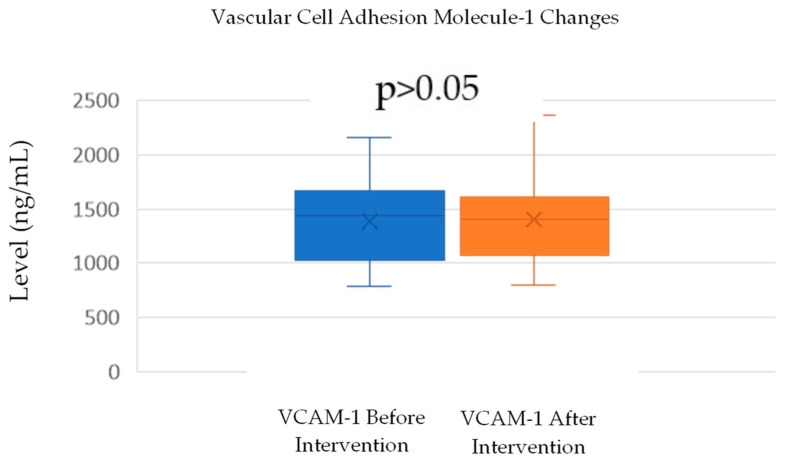
Change in VCAM-1 levels.

This study conducted correlation analyses to evaluate the relationships between VCAM-1 and TGF-β levels with the TCNS before and after the intervention. For VCAM-1 levels measured before the intervention, there was a weak positive correlation with TCNSs before (r = 0.117; *p* = 0.277) and after the intervention (r = 0.125; *p* = 0.263); however, these correlations were not statistically significant. A statistically significant moderate negative correlation was observed between VCAM-1 levels before the intervention and TGF-β levels before the intervention (r = −0.338; *p* = 0.039), indicating that higher VCAM-1 levels were associated with lower TGF-β levels prior to the intervention. VCAM-1 levels after the intervention showed a weak, non-significant positive correlation with TCNSs both before (r = 0.264; *p* = 0.087) and after the intervention (r = 0.262; *p* = 0.089). Notably, VCAM-1 levels after the intervention were significantly negatively correlated with TGF-β levels both before (r = −0.521; *p* = 0.002) and after the intervention (r = −0.397; *p* = 0.018), suggesting an inverse relationship between these biomarkers (Table 5; Figure 5).

**Table 5:** Pearson’s correlation test of variables.

	TCNS Before		TCNS After		TGF-β Before		TGF-β After	
	Correlation	*p*-Value	Correlation	*p*-Value	Correlation	*p*-Value	Correlation	*p*-Value
VCAM-1 before	0.117	0.277	0.125	0.263	−0.338	**0.039**	−0.197	0.158
VCAM-1 after	0.264	0.87	0.262	0.089	−0.521	**0.002**	−0.397	**0.018**
TGF-β before	−0.300	0.061	−0.326	**0.045**				
TGF-β after	−0.320	**0.048**	−0.353	**0.033**				

Variables were measured before and at 4 weeks after DC administration. Abbreviations: Toronto Clinical Neuropathy Score (TCNS); Transforming Growth Factor-β (TGF-β); Vascular Cell Adhesion Molecule-1 (VCAM-1). The bold is to highlight the significant *p*-values.

TGF-β levels before the intervention showed a moderate negative correlation with TCNSs before the intervention (r = −0.300; *p* = 0.061), although this correlation did not reach statistical significance. However, there was a statistically significant negative correlation between TGF-β levels before the intervention and TCNSs after the intervention (r = −0.326; *p* = 0.045), suggesting that higher baseline TGF-β levels were associated with lower neuropathy symptoms after the intervention. TGF-β levels after the intervention also showed statistically significant negative correlations with TCNSs both before (r = −0.320; *p* = 0.048) and after the intervention (r = −0.353; *p* = 0.033), indicating that higher TGF-β levels following the intervention were associated with lower TCNSs, reflecting reduced neuropathy symptoms (Table 5).

**Figure 6:**
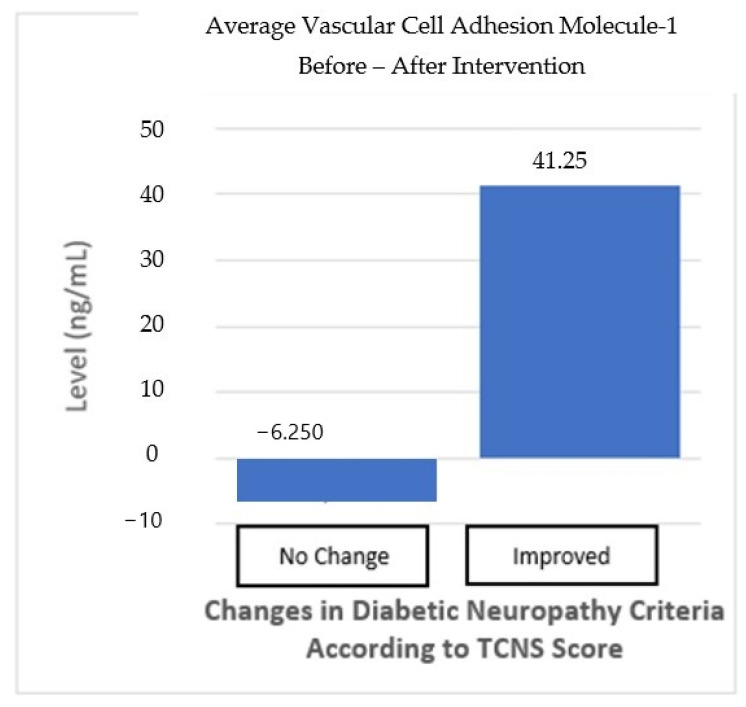
VCAM-1 average before and after the intervention based on improvements in the neuropathy criteria.

The subgroup analysis divided the subjects into two groups: one group with “no change” in the neuropathy criteria and one group with “improved” neuropathy criteria based on the TCNS. The “no change” in neuropathy group, as defined by the TCNS criteria, did not change from before and after the intervention, despite the TCNS reduction. In the “no-change” in neuropathy group, mean TGF-β levels increased by 0.821 ng/mL, but this was not statistically significant (*p* > 0.05). Meanwhile, the “improved” neuropathy group showed an average increase in TGF-β levels of 5.941 ng/mL (*p* > 0.05), indicating a trend toward more pronounced changes, even though this was not statistically significant. The difference between the two groups also shows no statistically significant result (*p* > 0.05) (Table 6; Figure 6).

**Table 6:** Subgroup analysis of biomarker changes based on improvements in the neuropathy criteria.

Diabetic Neuropathy * Criteria	Mean TGF-β Change (SD)	*p*-Value ^1^	*p*-Value ^2^	Mean VCAM-1 Change (SD)	*p*-Value ^1^	*p*-Value ^2^
No Change *n* = 16	0.821 (8.1)	0.692	0.158	−6.25 (329.7)	0.941	0.733
Improved *n* = 12	5.941 (49.8)	0.076		41.25 (399)	0.727	

Variables were measured before and at 4 weeks after DC administration. Abbreviations: Transforming Growth Factor-β (TGF-β); Vascular Cell Adhesion Molecule-1 (VCAM-1). * Based on TCNS Score. ^1^ Paired *T*-test. ^2^ Independent Sample *T*-test between 2 groups.

**Figure 7:**
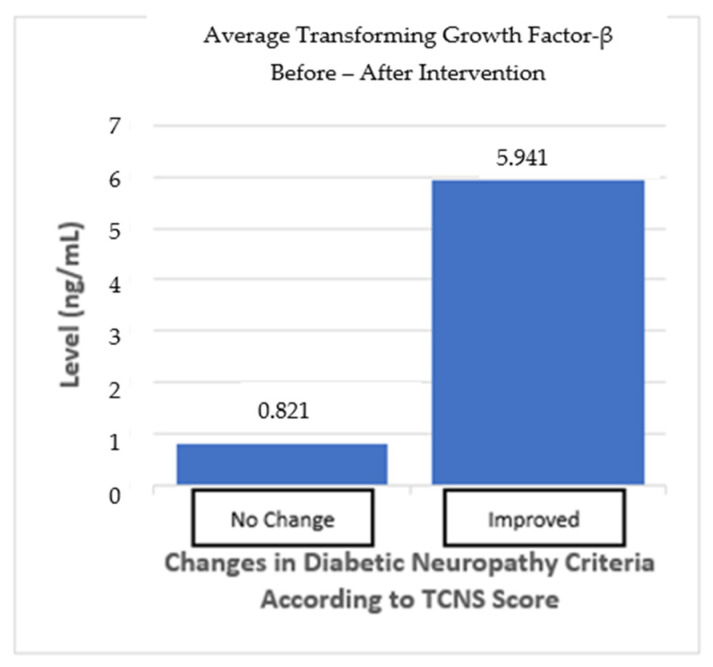
TGF-β average from before and after the intervention based on improvements in the neuropathy criteria.

In addition, changes in VCAM-1 levels were also analyzed for both groups. In the “no-change” in neuropathy group, VCAM-1 levels decreased by 6.25 ng/mL, but this decrease was not statistically significant (*p* > 0.05). In contrast, in the “improved” neuropathy group, there was an increase in VCAM-1 levels of 41.25 ng/mL, but this increase was also not significant (*p* > 0.05). The difference between the two groups also shows no statistically significant result (*p* > 0.05) (Table 6, Figure 7). Although the changes in TGF-β and VCAM-1 levels showed an improvement trend in the “improved” neuropathy group, these results were not statistically significant enough to be considered a definite effect of the intervention.

## 4. Discussion

DCs serve as professional antigen-presenting cells that bridge innate and adaptive immunity. They can promote antigen-specific tolerance by inducing regulatory T-cells and suppressing pro-inflammatory responses [14]. This has been demonstrated in systemic lupus erythematosus (SLE), where autologous DC therapy led to significant clinical improvements and reductions in inflammatory markers [15]. The potential for using tolerogenic DCs in autoimmune diseases is highlighted by their capacity to reset immune dysregulation, offering long-term therapeutic effects without relying heavily on immunosuppressants [14,15].

In infectious disease contexts, autologous DCs have been applied to elicit targeted immune responses, as seen in SARS-CoV-2 vaccine trials [16,17,18]. These studies showed that ex vivo-primed DCs can effectively activate T-cells, reduce systemic inflammation, and provide sustained immunity. This reflects their broader utility in managing chronic inflammatory states, including those involved in diabetic neuropathy.

Autologous dendritic cells represent a versatile tool in anti-inflammatory therapy. They offer a pathway for modulating immune activity, reducing inflammation, and potentially improving conditions like diabetic neuropathy through targeted, immune-regulating mechanisms.

This study showed a decrease in the mean TCNS after autologous dendritic cell administration. This decrease in the score reflects the improvement in DN symptoms experienced by patients. Research on diabetic mice showed similar results [19,20,21,22]. Natural anti-inflammatory agents have shown significant potential in managing diabetic neuropathy through various mechanisms. They reduce pro-inflammatory cytokines, oxidative stress, and gliosis while enhancing neurotrophic factors like brain-derived neurotrophic factor (BDNF), insulin-like growth factor-1 (IGF-1), and nerve growth factor (NGF). These agents modulate pathways such as those of AMP-activated protein kinase (AMPK) and PPAR-γ, contributing to neuroprotection [19,20]. Additionally, they improve nerve function and alleviate inflammation by suppressing markers like TNF-α and IL-6, underscoring their promise in therapeutic applications for diabetic neuropathy [22,23].

Using additional anti-inflammatory agents also can reduce nerve hypersensitivity to mechanical and temperature stimuli better than by using insulin alone [24]. Studies have also shown that this anti-inflammatory effect modulates gut microbiota. The study showed that the increased production of short-chain fatty acids, such as butyrate, produced by certain gut bacteria, can strengthen the integrity of the gut barrier and reduce its permeability. This contributes to decreased levels of endotoxins such as lipopolysaccharide (LPS) in the bloodstream, significantly lowering the systemic inflammatory response. Reduced levels of pro-inflammatory cytokines such as TNF-α and IL-6 following gut microbiota intervention have also been shown to correlate with improved symptoms of diabetic neuropathy, including reduced pain and improved peripheral nerve function [25].

TGF-β plays a vital role in tissue repair and immunomodulation. Broader evidence shows that TGF-β plays an essential role in nerve repair and regeneration, especially after immunomodulating therapies such as the introduction of autologous dendritic cells. Echeverry et al. showed that TGF-β1 effectively reduced neuropathic pain in a mouse model by inhibiting neuroinflammation, preventing neuronal damage, and suppressing microglia and astrocyte cell activation, thereby reversing the conditions of mechanical allodynia and thermal hyperalgesia [26]. This study supports evidence of TGF-β’s important neuroprotective and anti-inflammatory role in reducing neuropathic pain, suggesting its potential as a therapeutic target for treating various neuropathic conditions.

Clinically, these findings confirm that TGF-β may have an essential role in improving neuropathic conditions, especially after intervention. In the group of patients who experienced improvement in DN criteria, there was a more significant increase in TGF-β levels, although this was not statistically significant. As a growth factor involved in tissue repair processes and modulation of inflammatory responses, TGF-β may have a protective effect in peripheral neuropathy [27]. Although the correlations after intervention were insignificant, the results after intervention showed that TGF-β levels were associated with decreased TCNSs, indicating clinical improvement in patients. Similar results were reported by Ye et al., indicating that TGF-β has a strong correlation with the process of nerve regeneration. TGF-β not only helps facilitate debris clearance and the establishment of a supportive microenvironment but also directly increases the capacity of neurons to regrow [28]. As such, TGF-β is considered an essential factor in facilitating peripheral nerve recovery after injury [29].

In addition, there was a significant negative correlation between TGF-β and VCAM-1 levels, with moderate correlation strength. This is in line with the findings of Park et al., who stated that TGF-β and VCAM-1 have a relationship in which TGF-β acts as a regulator that suppresses VCAM-1 expression [30], which is usually induced by pro-inflammatory cytokines such as IL-1β and TNF-α in endothelial cells [31]. This study shows that TGF-β levels are inversely correlated with VCAM-1-expression, which is essential for recruiting immune cells to inflamed tissues. By suppressing the expression of VCAM-1, TGF-β1 plays a role in controlling the inflammatory response in endothelial cells, which could be very beneficial in inflammation control [31]. Furthermore, a study indicates that during peripheral nerve regeneration, TGF-β levels increase, peaking for approximately seven days. Therefore, the recommended evaluation after therapy should occur weekly for the first month [29].

Vascular Cell Adhesion Molecule-1 (VCAM-1) is critical in the pathophysiology of DN, serving as a mediator of inflammation and endothelial dysfunction. Hyperglycemia and oxidative stress in diabetes upregulate VCAM-1 expression, facilitating leukocyte adhesion to the endothelium and amplifying inflammation [32,33]. This contributes to nerve damage and the progression of DN. Elevated VCAM-1 levels correlate with the severity of diabetic complications, including neuropathy, nephropathy, and retinopathy [34]. The interaction of VCAM-1 with cytokines like TNF-α and IL-1 activates NF-κB signaling, further exacerbating inflammatory responses [35].

Those findings show some discrepancies with our study. This suggests that VCAM-1 levels may not always directly correlate with clinical improvements in diabetic neuropathy. The observed increase in VCAM-1 might be attributed to persistent hyperglycemia, leading to the formation of advanced glycation end products (AGEs), or, potentially, be due to insulin resistance [36,37]. Notably, our study showed no significant difference in fasting blood glucose (FBG) levels after treatment, which could indicate that hyperglycemia-induced oxidative stress or insulin resistance might be driving the increased VCAM-1 expression, independently of clinical improvements.

This study showed no significant changes in the subjects’ fasting blood glucose levels before and after autologous dendritic cell administration. However, there was an improvement in DN symptoms, as indicated by a significant improvement in the TCNS. The improvement was also correlated with increased TGF- β levels as a marker of an enhanced anti-inflammatory response after the autologous dendritic cell administration. Although glycemic control is essential for managing diabetes, it is often insufficient to prevent or slow the progression of DN in T2DM. Strict glycemic control is also more effective in alleviating DN symptoms in Type 1 diabetes mellitus (T1DM) than in T2DM, which is still unclear [38]. DN can develop even in newly diagnosed patients, suggesting that factors other than hyperglycemia, such as oxidative stress and inflammation, play a role in nerve damage [39].

This study has several limitations. The small sample size and short follow-up period limit the generalizability and long-term applicability of the findings. The lack of a randomized control group prevents definitive conclusions about the intervention’s efficacy, and conducting the study at a single center may reduce its representativeness. Additionally, the analysis was limited to a few biomarkers, leaving the broader mechanisms of action unexplored, and the study did not assess the effects of repeated or combined interventions.

## 5. Conclusions

This study shows that administering autologous dendritic cells significantly improves neuropathy symptoms by substantially decreasing the TCNS, reflecting improved DN symptoms. Although there was a trend toward increased VCAM-1 and TGF-β levels, these changes were not statistically significant. However, these findings indicate that autologous dendritic cells might exert anti-inflammatory effects and support nerve regeneration. The significant negative correlation between TGF-β and VCAM-1 corroborates the role of TGF-β in suppressing the expression of inflammatory molecules, reducing inflammation, and in promoting nerve repair.

In addition, research results show that glycemic control alone is not enough to prevent or improve diabetic neuropathy effectively. Other factors, such as inflammation and oxidative stress, play a role in the development of DN, even in patients with controlled glucose levels. Through their immune modulation and anti-inflammatory role, autologous dendritic cells show potential as adjunctive therapies for treating diabetic neuropathy. Thus, although further studies are needed to validate these findings, this study suggests that autologous dendritic-cell-based therapy may be a promising complementary approach for managing diabetic neuropathy through inflammation amelioration and nerve regeneration.

Future studies should include larger multicenter cohorts with longer follow-up periods and randomized controlled designs. Incorporating additional biomarkers and evaluating patient-reported outcomes will also enhance the robustness and clinical relevance of these findings.

## Data Availability

All data are available upon request.
